# Soil Water Balance and Water Use Efficiency of Dryland Wheat in Different Precipitation Years in Response to Green Manure Approach

**DOI:** 10.1038/srep26856

**Published:** 2016-05-26

**Authors:** Dabin Zhang, Pengwei Yao, Zhao Na, Weidong Cao, Suiqi Zhang, Yangyang Li, Yajun Gao

**Affiliations:** 1 College of Natural Resources and Environment, Northwest A&F University, 712100, Yangling, Shaanxi, China; 2 Bayannaoer Academy of Agricultural and Animal Sciences, 015000, Bayannaoer, Inner Mongolia, China; 3 Institute of Agricultural Resources and Regional Planning, Chinese Academy of Agricultural Sciences, 100081, Beijing, China; 4 Institute of Soil and Water Conservation, CAS & MWR, 712100, Yangling, Shaanxi, China; 5 Key Laboratory of Plant Nutrition and Agri-environment in Northwest China, Ministry of Agriculture, 712100, Yangling, Shaanxi, China.

## Abstract

Winter wheat (*Triticum aestivum* L.) monoculture is conventionally cultivated followed by two to three months of summer fallow in the Loess Plateau. To develop a sustainable cropping system, we conducted a six-year field experiment to investigate the effect of leguminous green manure (LGM) instead of bare fallow on the yield and water use efficiency (WUE) of winter wheat and the soil water balance (SWB) in different precipitation years in a semi-arid region of northwest China. Results confirmed that planting LGM crop consumes soil water in the fallow season can bring varied effects to the subsequent wheat. The effect is positive or neutral when the annual precipitation is adequate, so that there is no significant reduction in the soil water supplied to wheat. If this is not the case, the effect is negative. On average, the LGM crop increased wheat yield and WUE by 13% and 28%, respectively, and had considerable potential for maintaining the SWB (0–200 cm) compared with fallow management. In conclusion, cultivation of the LGM crop is a better option than fallow to improve the productivity and WUE of the next crop and maintain the soil water balance in the normal and wet years in the Loess Plateau.

Water deficiency is the main obstacle to the primary production in the dryland regions^1^. Natural rainfall is the sole water resource for most of the farmland in the Loess Plateau^2^. Most of the annual precipitation (50–60%) occurs from June to September^3^. Due to the large variation in the inter-annual precipitation, extensive soil erosion caused by wind or/and water is common in this typical dryland area. Meanwhile, unreasonable cultivation practices, including the shortage of organic fertilizer input and intensive soil cultivation, exacerbate the soil quality degradation, resulting in soil with a more fragile physical structure and low organic matter content (two-thirds of this region is below 1.0%)^4^. With the rapid development of intensive agriculture, crop production in dryland regions gradually faces the challenge of a scant water supply together with a nutrient deficit, not only in China but also in other countries^1^. Developing a sustainable cropping system to effectively improve crop productivity and water use efficiency and to maintain water balance is urgent for dryland farming in the Loess Plateau.

Winter wheat is one of the main food crops in the Loess Plateau. The cultivation area is nearly 4.3 million hectares, accounting for 40% of the food crops^5^. In this region, winter wheat monoculture is a common practice, followed by more than three months of summer fallow each year. The area of bare land during the summer fallow period is greater than two million hectares, accounting for nearly forty percent of the cultivated land in this dryland region^6^. The wheat yield depends not only on the growing season precipitation but also on the amount of water stored in the soil in the fallow season in the dryland cropping systems^7^,^8^. Mainly due to the large variations in both the annual and inter-annual precipitation amounts, the wheat yields have been unstable for a long time, averaging 2.5 to 3.7 t/ha, which presents a substantial difference compared with the average yields in China (4.7 t/ha) and some European countries (averaging 6 to 7 t/ha)^9^,^10^. Moreover, summer fallow, a traditional cultivation measure whose original purpose was to store more water in the soil during the rainy season, results in a low utilization efficiency of natural resources^1^. The fallow efficiency under bare fallow is low, accounting for an average of 28% of the fallow precipitation^11^. Furthermore, bare fallow may lead to further degradation of the fertilized soil due to water or/and wind erosion, which poses another great threat to sustainable production. Therefore, it is critical to pursue methods to effectively use the natural resources during the rainy season to prevent the soil fertility from further degradation and to improve the wheat productivity and WUE in this dryland region.

Application of green manure in an agro-ecosystem is an alternative method to establish a sustainable cropping system^12^. Green manure, which is primarily used as a soil amendment and an added nutrient for subsequent crops, is a cleaner and more secure organic fertilizer source that has been the essence of traditional agriculture in ancient China^13^. Due to their unique ability to fix atmospheric N_2_ via root nodules and produce substantial biomass and nutrient accumulations, the legume green manure (LGM) has become one of the most common and widely studied crop in the worldwide various agro-ecosystems. It has been demonstrated in different regions of the world that LGMs within a cropping system can (i) improve the grain yield of the subsequent cereal crops^14^,^15^,^16^, (ii) provide net additions of N^17^,^18^, maintain soil fertility and biological activity^19^, and (iii) reduce N fertilizer application needs^20^,^21^. Consequently, growing the LGMs during the summer fallow period and incorporating them into the soil at a suitable stage is a viable method to maintain the nutrients, soil structure, and subsequent crop growth and yield. However, it should be noted that the application of cover crops (including LGMs) can have negative effects on the soil water supply for the next crop due to the extra water depletion during its growth, especially when the precipitation is limited and highly erratic, as in semi-arid and arid regions^22^. Vigil and Nielsen^22^ and Nielsen and Vigil^23^, who replaced a portion of the fallow period with legumes in winter wheat–fallow systems in the central Great Plains, USA, observed that the wheat yield decreased substantially following the legumes. After testing different crop rotation systems, Li *et al.*^24^ showed somewhat inconsistent results. They reported that growing a crop for forage during the fallow period does not greatly influence the quantity of water stored in the soil to be used by the subsequent winter wheat in the middle-west Loess Plateau. Therefore, it is essential to investigate the effect of the LGM crop on the water use, productivity and WUE of winter wheat, as well as the soil water balance in different precipitation years in the Loess Plateau of China.

For this purpose, we explored the temporal characteristics of precipitation (e.g., the decadal precipitation trend and seasonal distribution) for the winter wheat zone in the Loess Plateau, using 57 years of precipitation data. We also identified the impacts of the LGM crop on the subsequent wheat yield, WUE, and soil water balance in dry, normal, and wet years. The results of this study will be used to establish a LGM-based cropping system, providing a theoretical basis and guiding field management strategies for local and other similar dryland regions.

## Materials and Methods

### Experimental setup

A six-year field experiment was conducted at the Station of the Agricultural Technology Demonstration Center of Changwu County, Shaanxi Province (35°12′N, 107°44′E, with an altitude of 1,220 m), on the southern Loess Plateau, beginning in June 2008. The experimental site has a semi-arid and continental monsoon climate with an average annual sunlight of 2,230 hours and an average open pan evaporation of 1,440 mm, measured from 1957 to 2009^25^. The average annual temperature is 9.1 °C, with typically 171 frost-free days each year. Agricultural production in this region is completely dependent on the natural precipitation, with 50–60% of the annual rainfall occurring from June to September. The long-term (1958–2014) mean annual, summer fallow season, and wheat growing season rainfall in the experimental site was 578.9, 315.4, and 263.5 mm, respectively.

The soil at the study site is classified as aridic and loamy, Cumulic Haplustoll^26^, with moderate fertility and high permeability, which developed from loess deposits. The soil pH, organic matter, total N, total P, mineral N, available P, available K, and the field capacity at the 0 to 20 cm depth were 8.11, 12.0 g/kg, 0.79 g/kg, 0.66 g/kg, 13.7 mg/kg, 24.6 mg/kg, 161 mg/kg, and 22.4%, respectively, in June 2008. Prior to this experiment, the field had been cultivated with winter wheat for many years.

The experiment was arranged in a complete split-block design. The four main treatments were (1) summer fallow―winter wheat (FW, as the control), (2) *Huai* bean (*Glycine ussuriensis Regel et Maack.*)―winter wheat (HW), (3) soybean [*G. max* (L.) *Merr.*]―winter wheat (SW), and (4) mung bean (*Phaseolus radiatus* L.)―winter wheat (MW). The subplot treatments were N fertilizer rates applied in the wheat growth season: 0 (N0), 108 (N108, 67% of N162), 135 (N135, 83% of N162), and 162 kg N/ha (N162, which was the typical rate of N fertilizer application in the study area). The main plots were 6 × 20 m with a boundary of 0.3 m between each plot, and the subplots were 6 × 5 m. Each main treatment had three replications. Results of the N108 and N135 plots were not exhibited in this study.

### Field management

The LGMs were sown from late June to early July and were terminated at approximately full bloom from the end of August to early September each year. The plots with mung bean, soybean, and Huai bean were seeded with a row seeder at seeding rates of 135, 150, and 165 kg/ha, respectively. To increase the aboveground biomass and nutrient accumulation, P fertilizer was added as triple superphosphate (TSP, 46% P_2_O_5_) at a rate of 40 kg/ha before planting in 2008 and 2009. After seven or eight weeks, the fresh aboveground biomass of LGMs was manually harvested from the entire plot and weighed. The aboveground portions of the legumes were cut into approximately 5-cm pieces using the blade of a local farmer and were immediately incorporated into the soil to a depth of 20 cm using a rotary tiller. After the LGMs decomposed for two to three weeks, winter wheat (Changwu 521) was planted at a seeding rate of 180 kg/ha with varying amounts of N fertilizer (urea, 46% N) and 120 kg P_2_O_5_/ha (TSP, 46% P_2_O_5_) for each treatment in late September. After harvesting the wheat in late June, the field was plowed with a rotary tiller to prepare the LGM seedbed. Wheat stubble was removed from the plots. Field management, including pest and weed control, were performed according to the local farming practices.

### Field and laboratory measurements

The winter wheat yield was recorded by manually harvesting from a net plot area of 10 m^2^ in late June. The wheat samples, which were taken from 4 × 1-m-long randomized rows of each plot, were dried at 95 °C for 0.5 h and at 65 °C for 24 h until a constant weight was achieved. The soil bulk density at the depth of 0–200 cm was measured in 2008 using the cutting ring method^27^. To test the soil moisture content, one or two soil samples in each plot were collected with an auger from 0–200 cm in 20-cm increments at the times of the winter wheat harvesting in each year. Soil samples at the depth of 0–200 cm were collected from 16 specific plots of the experiment (e.g. all the N0 experimental plots) at the times of wheat planting in each year. All soil samples were immediately weighed and placed in a drying oven at 105 °C for 24 h until a constant weight was achieved. Precipitation data (including snow during the winter months) was measured using a rain gauge approximately 500 m far from the experimental site.

### Data analysis

According to the periods of winter wheat growth, the fallow season precipitation (FSP) is identified as the precipitation that occurred from July to September, which is the summer fallow period for the winter wheat monoculture system in the experimental site. The growing season precipitation (GSP) is identified as the precipitation that occurred from October to June, which is the growing period for winter wheat. Hence, the annual precipitation was calculated as the sum of the precipitation from July to June, i.e., FSP plus GSP^25^.

The amount of soil water at the times of wheat planting (SWP) and harvesting (SWH) was expressed as the water stored in the 0–200 cm soil depth. Soil water depletion during the wheat growing season (SWD) was calculated as the difference between SWH (end of growing season) and SWP (beginning of growing season), whereas soil water storage during the fallow period (SWS) was taken as the difference between SWP (end of fallow) and SWH (beginning of fallow). Hence, the soil water balance (SWB) was calculated as the difference between SWD and SWS^28^.

Seasonal evapotranspiration (ET) was calculated using the soil water balance equation in each growing season for each year of the study as follows^29^


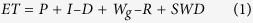


where *ET* is the evapotranspiration (mm); *P* the total seasonal precipitation (mm); *I* the amount of irrigation (mm); *D* the soil water drainage (mm); *W*_*g*_ the amount of water used by the crop through capillary rise from groundwater (mm); *R* the surface runoff; and *SWD* the change of soil water content from planting to harvest in the measured soil depth (mm). When the groundwater level is more than 4 m below the ground surface, the capillary rising of groundwater is considered negligible. The irrigation and runoff are also ignored because there is usually limited water to irrigate and no runoff in the flat farmland of the Loess Plateau. Based on the infrequent frequency of extreme high rainfall occurred in this dryland region, the probability of soil water drainage below the measurement depth was not considered in the current research. Therefore, *I*, *D*, *W*_*g*_, and *R* were regarded as zero in this experimental condition.

Water use efficiency by crop was calculated as follows^11^


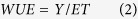


where *WUE* is the water use efficiency (kg/m^3^); *Y* the grain yield; and *ET* the evapotranspiration (mm), which is calculated from Eq. (1).

Similar to WUE, the water productivity (WP), expressed as the dry biomass of summer legumes per unit ET, was calculated for the LGMs^30^.

The fallow precipitation storage efficiency (PSE) was calculated using the following equation^31^


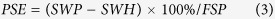


### Statistical analysis

The analysis of variance (ANOVA) for the complete split-block design was performed to determine the LGM, N fertilizer (N), and interactive effects of treatments and year (Y) on the parameters using SAS software^32^ (Table 1). A mixed model with the LGM and N as the fixed effects and year as the random effect was applied to check the significance of the main factors (LGM and N or Y), of their interactions, and a means comparison using Duncan’s test. A one-way ANOVA was applied to check the significance of the treatments in each year and a means comparison using Duncan’s test. A linear regression analysis was performed to predict the decadal-mean precipitation trends during the 57 years. A multiple regression analysis was performed to assess the probability of precipitation throughout the annual year, the FSP, and GSP. A correlation analysis was conducted to contrast the relationships between the annual precipitation, FSP, and GSP and the wheat grain yield for the LGM treatments. The *P* value at significance levels of 0.05 and 0.01 was marked with one asterisk and two asterisks, respectively, whereas the case of “not significant” was expressed as NS.

## Results

### Characteristics of precipitation

**Figure 1:**
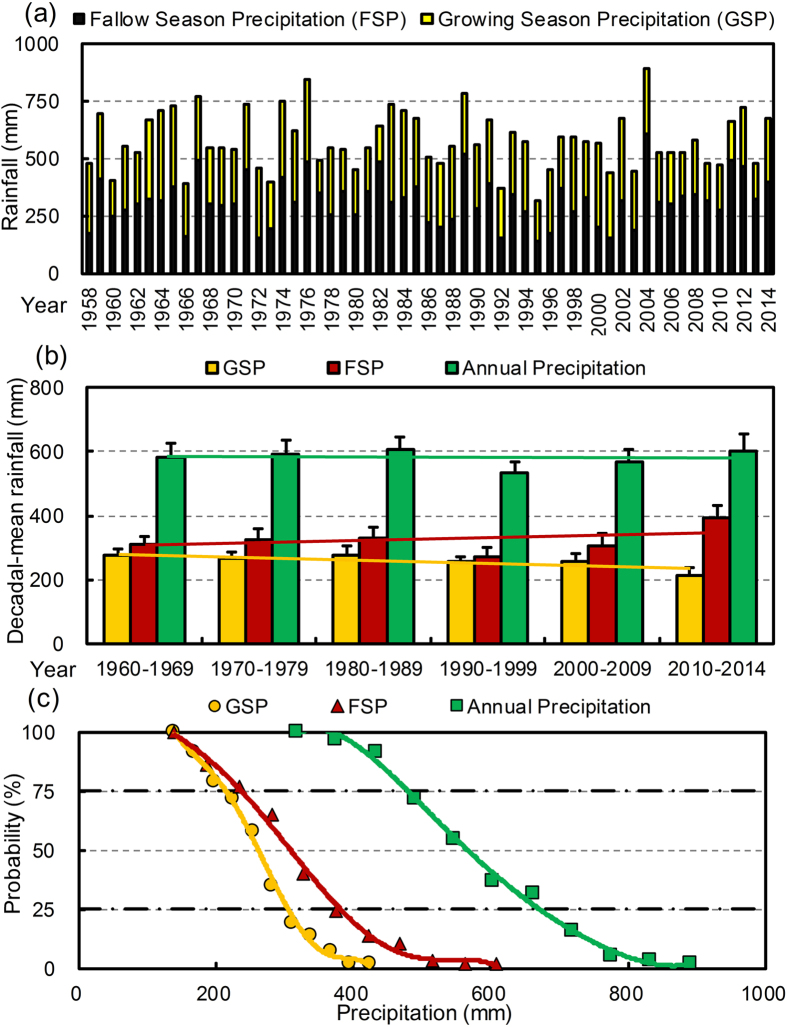
Summer fallow season precipitation (FSP) and wheat growing season precipitation (GSP) (**a**), the decadal-mean precipitation (**b**), and the probability of precipitation throughout the annual year, FSP, and GSP (**c**) at the Changwu County Meteorological Station from 1958 to 2014. The bars in (**b**) show the standard errors. The solid heavy lines in (**c**) represent the probabilities of 25% and 75% for precipitation.

The long-term annual precipitation measured at the Changwu County Meteorological Station ranged from 318 (1995) to 891 mm (2004), with a 57-year average of 578.9 mm and a coefficient of variation (CV) of 21% (Fig. 1a). The GSP ranged from 140 (1977) to 425 mm (1983), with an average of 263.5 mm and a CV of 25%. The average FSP was 19% greater than the GSP, with a wider range (140–609 mm) and a higher variability (33%). The slope of the decadal-mean GSP versus time was significantly different to zero (−10.08), however, there were no significant differences between the slopes of the decadal-mean FSP (8.32) as well as the annual precipitation (−1.76) versus time and zero (Fig. 1b). The annual mean precipitations in the 1960s, 1970s, 1980s, 1990s, 2000s, and 2010–14 were 584.6, 591.7, 607.4, 531.7, 565.5, and 603.2 mm, respectively.

From the analysis of the probability of annual year precipitation from 1958 to 2014, three types of normal, dry, and wet years were obtained (Fig. 1c). The precipitations with exceedance probabilities of 25% and 75% for a single year were 671 and 481.1 mm, respectively. Compared with the normal years (with precipitation ranged from 481.1 to 671 mm), the annual mean precipitation in dry years (less than 481.1 mm) was decreased by 24%, whereas that in wet years (more than 671 mm) increased by 30%. Based on the probabilities of GSP and FSP, three types of normal, dry, and wet seasons were also distinguished. Precipitations with exceedance probabilities of 25% and 75% for the winter wheat growing season were 305.2 and 215 mm, respectively. The corresponding precipitations for the summer fallow season were 381.4 and 237.6 mm, respectively.

During the test years, the annual precipitations in 2008–09, 2009–10, 2010–11, 2011–12, 2012–13, and 2013–14 were 480.5, 474.5, 664.5, 722.2, 476.8, and 677.9 mm, respectively. According to the annual precipitation, dry years occurred in 2008–09, 2009–10, and 2012–13, accounting for 50% of the six years. Wet years occurred in 2011–12 and 2013–14, accounting for 33.3% of the experimental years. The only one normal year occurred in 2010–11. The frequencies of normal, dry, and wet seasons were not consistent with those of the three corresponding types of the past 57 years. There was no dry fallow season or wet growing season during the experimental years. Normal fallow seasons occurred in 2008–09, 2009–10, and 2012–13, and wet seasons occurred in the other three years. Except for 2011–12 and 2013–14, the other four years experienced dry wheat growing seasons, accounting for 66.7% of the study years.

### ET, WP, and PSE in the summer fallow season

The ET, WP, and PSE during the summer fallow season varied significantly from year to year. However, they were not influenced by LGM (*P* > 0.05) (Table 1). There was no linear relationship between the annual precipitation and the ET and WP of the fallow season. The mean ET in the normal years was the highest, 25% and 31% greater (*P* < 0.01) than those in the dry and wet years, respectively. Moreover, it was 7.7% greater (*P* > 0.05) in the dry years than in the wet years. The mean WP was the highest in the dry years (1.07 kg/m^3^), the lowest in the normal years (0.94 kg/m^3^), and intermediate for the wet years (0.98 kg/m^3^). There appeared an obvious upward tendency in the PSE with the precipitation increase. The mean PSE in the wet years was 16% and 34% greater (*P* < 0.05) than those in the normal and dry years, respectively. In addition, it was also 22% greater (*P* > 0.05) in the normal years than that in the dry years (Table 2).

Growing Huai bean, soybean, and mung bean as the LGM crop during the fallow period resulted in significant increases of the seasonal ET (12–22%) in the dry years, whereas the ET in the LGM systems were comparable with or even less than that in the fallow system in the normal and wet years, mainly due to the increasing rainfall during the fallow period (Fig. 1). This indicates that the LGMs would not cause extra water use compared with the traditional bare fallow management in the normal and wet years. The PSE was 13–25% less (*P* < 0.05) for the LGM systems than that for the summer fallow system in the dry years because of the greater ET in the LGM systems. However, there was no significant difference in PSE between the LGM and FW systems in the normal and wet years. Growth of the LGMs increased the WP in the fallow season. Although the WP of the LGM treatments varied with the year, the mean WP of Huai bean during the six tested years was 6.4% and 15% greater than those of mung bean and soybean, which can be ascribed to its greater fresh biomass with a lower ET rate during the summer fallow season.

### Grain yield and WUE in the wheat growing season

The wheat grain yield, ET, and WUE were significantly affected by year and year × LGM (Table 1). Notably, there appeared a significant increase in the wheat yield as the annual precipitation increases. The mean yield in the wet years was 5,894 kg/ha with a CV of 14%, almost two times and 55% greater (*P* < 0.05) than those in the dry and normal years, respectively. Moreover, the yield in the normal years was 32% greater (*P* < 0.05) than that in the dry years. The mean ET was the highest in the wet years the lowest in the normal years, and intermediate for the dry years. Similar to the wheat yield, the mean WUE in the wet years was the highest, almost two times and 55% greater than those in the dry and normal years, respectively. In addition, it was also 33% greater (*P* < 0.05) in the normal years than that in the dry years.

Mainly due to the extra water consumption in the fallow season, the LGM crop decreased the wheat yield by 6.3–14% (*P* < 0.05) in the dry years compared with FW, with the highest for mung bean and the lowest for soybean. The opposite was true in the normal and wet years. The wheat yields for the LGM systems were increased by 19–39% (*P* < 0.05) and by 4.2–28% (*P* < 0.05) compared with those for the fallow system in the normal and wet years, respectively. Compared with FW in the wheat growing season, the ET of HW, SW, and MW were reduced by 8.3–14% in the dry years, by 2.1–9.1% in the normal years, and by 4.3–12% in the wet years, respectively (*P* < 0.05). Consequently, WUE was 4.8–5.7%, 33–62%, and 16–46% greater in the LGM systems than that in the fallow system in the dry, normal, and wet years, respectively, except for the MW in the dry years.

Application of N fertilizer not only increased the wheat yield and ET (*P* < 0.01), but also increased the WUE (*P* < 0.01) despite the different precipitation through the test years (Table 3). There was no obvious interaction effect between the LGM and N fertilizer treatments on the ET in the dry years or the yield, as well as the WUE in the normal and wet years.

**Figure 2:**
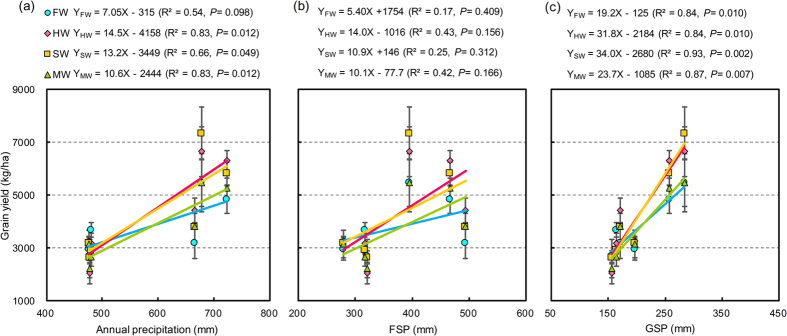
Relationships between annual precipitation (**a**), FSP (**b**), and GSP (**c**) with wheat grain yield under the LGM treatments from 2009 to 2014 (n = 288). The LGM treatments are as follows: FW for summer fallow-winter wheat (CK), HW for Huai bean-winter wheat, SW for soybean-winter wheat, and MW for mung bean-winter wheat. Each value is presented as the mean for the same LGM treatment each year. The bars show the standard errors (n = 12). Linear regression models and correlations between the different characteristics and the yield are presented in each upper corner. R^2^ corresponds to the correlation coefficient.

Correlation coefficients of the wheat grain yield with the annual precipitation, FSP, and GSP for the LGM systems are presented in Fig. 2. No apparent relationship was found between the FSP and yield. However, there was a significant positive correlation between the yield and the annual precipitation and GSP, indicating the key role of GSP, rather than FSP, in grain yield production under the rain-fed cropping system. For the regression of yield versus GSP, the slopes of the LGM systems (ranged from 23.7 to 34.1) are greater than those of the fallow system (19.2). Similar results can observed for the wheat yield versus the annual precipitation and FSP.

### Soil water status and balance

**Figure 3:**
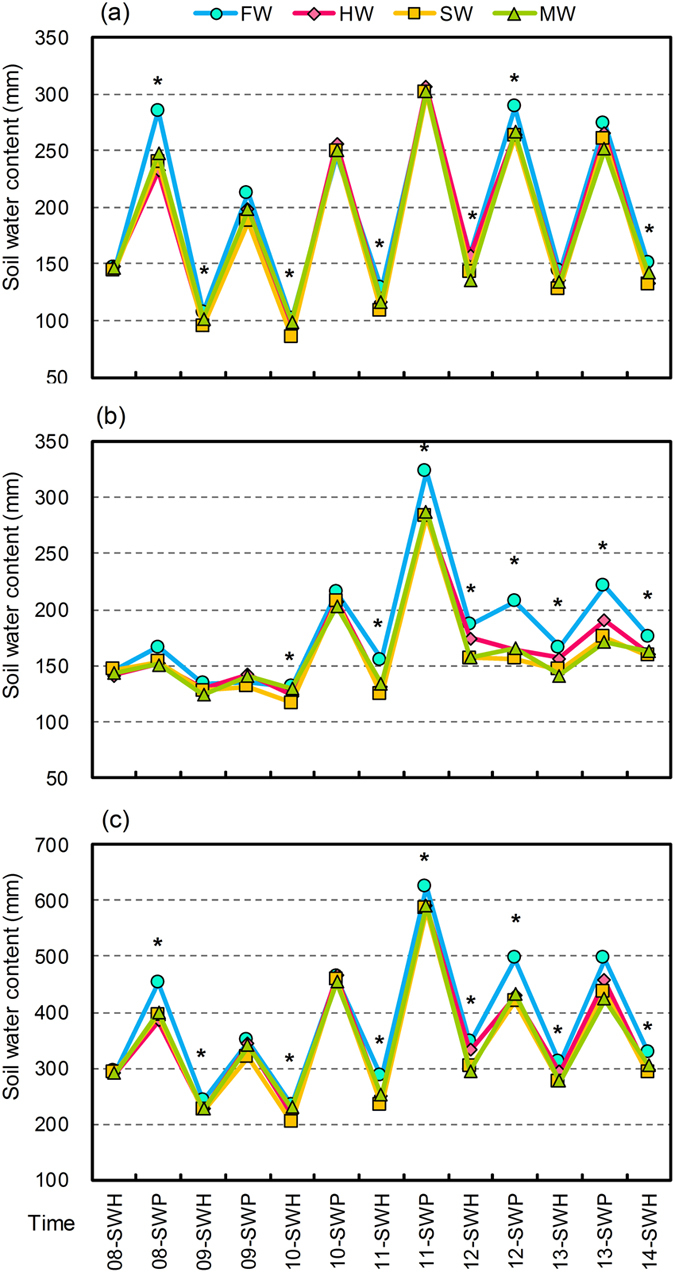
Distributions of soil water content (mm) in the 0–100 (**a**), 100–200 (**b**), and 0–200 cm (**c**) depths under the LGM treatments at the times of winter wheat planting and harvesting from 2008 to 2014. The LGM treatments are as follows: FW for summer fallow-winter wheat (CK), HW for Huai bean-winter wheat, SW for soybean-winter wheat, and MW for mung bean-winter wheat. SWP and SWH are the soil water contents at the times of the full bloom stage of winter wheat planting and harvesting, respectively. Each value is presented as the mean for the same LGM treatment. “*” indicates significant differences at *P* < 0.05 (Duncan’s test) between the LGM treatments.

The distribution of soil water in the 0–100, 100–200, and 0–200 cm depths at the times of winter wheat planting and harvesting from 2008 to 2014 showed that the water content in the upper 0–100 cm soil depth fluctuated more severely than that in the 100–200 cm depth (Fig. 3). At the times of wheat planting, the mean soil water contents in the 0–100 cm and 100–200 cm depths were 256 mm (CV, 18%) and 193 mm (CV, 28%), representing 56% and 44% of the total available water in the 0–200 cm soil depth, respectively. The mean water contents in the 0–100 cm and 100–200 cm soil depths decreased to 123 mm (CV, 18%) and 147 mm (CV, 13%), which represented 46% and 54%, respectively, of the 0–200 cm soil water depth at the times of harvesting.

The LGM approach significantly affects the water contents in the 0–100, 100–200, and 0–200 cm soil depths at the times of wheat planting and harvesting (Fig. 3). Compared with bare fallow, inclusion of the LGM crop in the traditional wheat cropping system decreased the mean soil water content at the depth of 0–200 cm by 47.6 mm (*P* < 0.05), by 3.2 mm (*P* < 0.05), and by 46.5 mm (*P* < 0.05) at the times of wheat planting in the dry, normal, and wet years, respectively. Overall, the mean soil water content at the depth of 0–200 cm under the LGM systems was 32.4 mm less than that in the FW system at the times of wheat planting, with 70% of the total water depletion occurring in the top 100 cm, and 30% in 100–200 cm. Similarly, the mean reductions in soil water content caused by the LGMs in the 0–200 cm depth were 20.4 mm (*P* < 0.05), 40.3 mm (*P* < 0.05), and 33.1 mm (*P* < 0.05) at the times of wheat harvesting in the dry, normal, and wet years, respectively. On average, the LGM approach decreased the soil water content by 28 mm compared to bare fallow in the 0–200 cm depth, with 43% of the total water depletion occurring in the top 100 cm, and 57% in 100–200 cm at the times of wheat harvesting during the test 6-years.

The SWS, SWD, and SWB in the 0–200 cm depth were significantly affected by the year. However, they were neither influenced by LGM nor year × LGM (*P* > 0.05) (Table 1). In the dry years, the SWS for the LGM systems were 11 to 19% (*P* < 0.05), 18 to 78% (*P* < 0.05), and 12 to 26% (*P* < 0.05) less than those for the fallow system in the 0–100, 100–200, and 0–200 cm soil depths during the fallow season, respectively. Despite the depth, there was no significant difference in the SWS between the LGM approach and the FW in the normal years. There were 0.3 to 5.9% and 3.5 to 5.8% (*P* > 0.05) increases in SWS for the LGM systems compared with those for the fallow system in the 0–100 and 0–200 cm depths in the wet years, respectively. A similar tendency in SWD during the growing season was found in the 0–200 cm soil depth. Based on SWB, the soil water in 0–200 cm was almost the same or greater for the LGM systems than for the bare fallow system in the normal and wet years, whereas soil water depletion was found in the upper depths in the dry years, which might bring negative effects on the growth and productivity of the subsequent crop in the rain-fed cropping system. On average, the LGM decreased the soil depletion by 13.3 mm (0–200 cm) during the growing season, while it decreased the soil water storage by 10.2 mm (0–200 cm) during the fallow season, thus increasing the soil water balance by 3.1 mm (0–200 cm) (*P* > 0.05) compared with conventional fallow.

## Discussion

### Soil water balance for the LGM systems

During the 6-yr period, we found that planting the LGM crop does not greatly affect the efficiency of precipitation storage and the quantity of water stored in the 0–200 cm soil depth during the summer fallow season in the normal and wet years (Table 2). Few differences in ET between the FW and LGM treatments suggest that the soil water evaporation rate of the fallow treatment was almost equal to the ET rate of the LGM treatments when the legumes were terminated at approximately full bloom, especially in the wet years. These findings are consistent with the results from other investigators^24^,^30^. Zhang *et al.*^3^ reported that there was no significant difference in the soil water storage level under catch cropping treatment compared with the conventional practice for all the three years. Moreover, the water balance and fallow efficiency under the green manure treatments would be similar to that of the conventional practice if the black bean (*Aphis faba*) were incorporated into the soil at a proper stage (45 days before sowing winter wheat)^33^. O’Dea *et al.*^34^ demonstrated in north-central Montana, USA, that green manure crops likely did not limit the soil water available to wheat, mainly resulting from the small soil water depletions in the green manure treatments below fallow at wheat seeding (17%; 30 mm) and near-record high rainfall during the wheat growing season (280–380 mm).

However, it should be noted that the application of the LGM approach could result in a further water depletion in a dry year with deficient rainfall or when termination date of fallow crops was late^30^,^33^. In this research, the ET (0–200 cm) was increased whereas PSE and SWS (0–200 cm) were decreased significantly under LGM than under FW in the dry years. This result is in accordance with the results presented by Zhang *et al.*^11^. They introduced the green manure crop in the conventional management in a 3-yr study, indicating that growing a green manure crop decreased the soil water storage at the times of wheat planting during a dry year than in a wet year. In rotations, deep-rooted legumes may use water at depth, thus obviating access by the subsequent crop and diminishing yield, especially in a year with low rainfall^35^.

Notably, the LGM approach increases the WP in the summer fallow season, suggesting that growing legumes, which produce high quantities of biomass, can achieve better natural resource use efficiencies. This is in line with Allen *et al.*^30^, who took a 12-yr study and found that the green manure crops, under proper management, maintains water productivity about three cropping cycles compared with traditional wheat–fallow rotations during the fallow period in the North America northern Great Plains.

According to the six-year results in the present study, the incorporation of the LGM crop had considerable potential for maintaining the soil water balance versus the conventional fallow approach in the normal and wet years (Table 4). Applying LGMs in the fallow season was an available option to keep the soil water balance because it adds an amount of high-quality organic material to the soil, thereby increasing the amount of soil organic matter (SOM) that could probably increase the porosity, infiltration and water-holding capability of the soil^36^. Similar with PSE and ET, there showed a strong negative influence in the soil water balance when LGMs were planted during the fallow season when the rainfall is not adequate (Fig. 3 and Table 4). This is consistent with some previous studies^22^,^33^. Furthermore, the LGMs reduced the downward water transport, leading to less deep percolation and lower amounts of water stored in the deeper soil profile^3^. Therefore, the specific climate condition should be considered when the LGM approach was applied to the water-limited agricultural system.

### Wheat grain yield for the LGM systems referring to precipitation

To better understand the patterns of the wheat yield and water use efficiency under the LGM system in different precipitation years, it is important to investigate the temporal characteristics of precipitation, such as the annual precipitation trends and seasonal distribution. In our study, the mean GSP, FSP, and annual precipitations during the 57-year period were comparable with the corresponding results stated in a report in the same region of the Loess Plateau^25^. According to the decadal-mean precipitations from 1960s to 2010–2014, there showed an obvious downward trend in the GSP (R^2^ = 0.628^*^) with an average decreasing rate of 2.5 mm/year in the study area. However, no apparent changes were observed in the annual year precipitation (R^2^ = 0.014^NS^) and FSP (R^2^ = 0.159^NS^) from 1958 to 2014 (Fig. 1a,b). This is in line with the results of a long-term (15-year) field experiment carried out in the Loess Plateau of China by Huang *et al.*^37^, who reported that there showed a marked decrease in the rainfall during the wheat growing season, however, no significant changes could be detected in the total rainfall from 1985 to 1999.

As the natural precipitation is the sole source of water in rain-fed cropping systems, winter wheat growth and productivity was closely related with the precipitation on the Loess Plateau of China^25^. In this study, the wheat yield was one to two times greater in the normal and wet years than that in the dry years, indicating that the crop productivity was significantly depressed in the years with low rainfall. There was no apparent relationship between FSP and yield, whereas there appeared an obvious positive correlation between yield and GSP, implying the key role of GSP, instead of FSP, in grain yield production under the rain-fed cropping system (Fig. 2). This result is consistent with Li *et al.*^24^, who found that the variation in the yield for wheat was not only associated with the pre-sowing soil water storage but also closely related to the growing season precipitation. According to the regressions of yield versus GSP, FSP, and annual precipitation, the greater slopes for the LGM systems than fallow system suggested that the wheat yield was more sensitive to the variations of the rainfall following the LGMs compared with bare fallow. In the dry years, the LGMs depressed the mean wheat yield by 9.8% (304 kg/ha) compared with bare fallow (Table 3), which may be related to the less precipitation in the growing season and extra water requirements of the LGMs during the summer fallow period. This result agrees with that presented by Vigil and Nielsen^38^ and Nielsen and Vigil^23^, who replaced a portion of the fallow period with legume production in the winter wheat–fallow systems in the central Great Plains, observing that the subsequent wheat yields substantially decreased following the legumes, mainly due to the extra water depletion caused by the cover crop growth. Unger and Vigil^22^ demonstrated that a consequence of growing cover crops (including green manure crops) is that they use soil water, which can have negative effects on the soil water supply and productivity for the next crop when the precipitation is limited and highly erratic, as in semiarid to arid regions.

Upon increasing the precipitation in the normal and wet years, there appeared a significant improvement in the wheat yield for the LGM systems (Table 3), suggesting that the positive effects of LGMs on the subsequent crop yield begun to occur when the natural rainfall is adequate under this dryland region. This is consistent with Zhang *et al.*^33^, who found that there appeared an increase in the following winter wheat yield under the green manure treatment compared with the conventional practice treatment during a relatively wet year. There were more visible beneficial effects of LGMs on the subsequent crop growth, production, and economic benefits in the wet years^21^. Previous studies also demonstrated that legumes could play a role in the maintenance of soil productivity in cropping systems through N_2_ fixation^18^, the recovery of soil nutrients^39^,^40^, and increases in the soil biological activity and microbial biomass accumulation^41^. In addition, these benefits to subsequent crops will persist^42^.

### ET and WUE of wheat for the LGM systems

Considerable variations in seasonal ET of wheat were recorded due to the large variation in the amount of precipitation received during the six years. Our six-year study found that growth of the LGM crop tended to decrease the ET in the wheat-growing season. This conflicts somewhat with the results of a short-term (three-year) field study in the semi-arid region of the northwest China by Li *et al.*^24^, who found that there showed a marked increase in evapotranspiration when fallow crops were added to the rotation cropping systems. Huang *et al.*^31^ also reported that involving peas in traditional winter wheat rotations markedly decreased the seasonal ET of wheat. The decrease in ET in the wheat-growing season for the LGM systems may be related with the improved soil fertility due to the inclusion of the fresh LGM biomass for continuous six years (data not shown). Applying the LGM approach was an available option to increase the amount of SOM, which could probably increase the porosity and water-holding capability of the soil^36^. Besides, Olness and Archer^43^ demonstrated that a 1% increase in soil organic carbon induce a 2–5% increase in the soil available water-holding capacity depending on the soil texture. Consequently, continuous inclusion of LGMs might have the positive effects on reducing the evapotranspiration compared with bare fallow system.

In rain-fed cropping systems, water for evapotranspiration was derived partly from soil water storage before wheat planting and partly from growing season precipitation. However, the relative contributions to ET from the two sources varied significantly with the annual precipitation and LGMs. In this trial, winter wheat for the LGM systems consumed 36–41% of the available water that came from soil water storage at planting for ET, whereas 59–64% came from growing season precipitation. For the bare fallow system, the mean available water consumed by wheat was 44% and 56% derived from soil water storage and growing season precipitation, respectively (Fig. 1 & Table 3). These results indicate that the application of the LGM approach has considerable potential for reducing the depletion of soil water storage and increasing the utilization efficiency of the growing season rainfall, thus helping to keep the water balance compared with bare fallow. One explanation about saving the soil water storage under the LGM systems compared with fallow system was probably related to the increased the porosity and water-holding capability of the soil due to the apparent improvements in SOM and soil bulk density over the six years (data not shown)^43^,^44^. Besides, the incorporation of high-quality organic material into the soil could not only enhance root growth but also improve crop water status and its tolerance to drought^45^, which may be favorable to alleviate water stress and improve the productive transpiration, especially in arid and semi-arid regions^28^.

WUE, the grain yield per unit of seasonal ET, varied greatly between LGM crops and the amount of precipitation received during the six years. The mean WUE of wheat grain in this trial (1.24 kg/m^3^) was close to the mean WUE value of wheat (1.20 kg/m^3^) in a 24-year fertilization study in the northeast part of the Loess Plateau^46^. Zhang *et al.*^47^ compiled a data base of 39 sets of experiments spanning 20 years and demonstrated that the average WUE value of winter wheat across all practices is 1.21 kg/m^3^ in the dryland area of the Loess Plateau of China. However, the value was 13% greater than the global average WUE of 1.09 kg/m^3^ for wheat^48^. With a greater grain yield in the normal and wet years, there was a significant increase in the WUE for the LGM systems over bare fallow. For the HW and SW systems, it is particularly noteworthy that the lowered grain yields in the dry years were not concomitant to substantially lowered WUE values. This is not in line with the reports presented by Zhang *et al.*^33^, who demonstrated that less available soil water for wheat transpiration caused an apparent decrease in the wheat yield and WUE for fallow crops versus conventional practice. Nielsen and Vigil^49^ stated that field pea (*Pisum sativum* L.) grown ahead of wheat did not improve the wheat yield and WUE compared with wheat preceded by wheat, proso millet (*Panicum miliaceum* L.), or fallow in the central Great Plains. Notably, the decrease in WUE under alternative practices can be attributed to the corresponding decrease in the grain yield, addressing the strong positive relationship between the WUE and yield^47^. Moreover, the inconsistent results in WUE for the LGM approach in the previous studies might be related to the short period of the field trials (less than three years) or the specific climate conditions with extremely low annual precipitation (<350 mm). Mainly due to their poor growth and low adaptabilities to the dryland condition, mung bean and soybean produced less biomass during the fallow period and led to greater reduction in wheat yield in the dry year than Huai bean. Cherr *et al.*^12^ reported that establishment of large-seeded LGMs such as soybean may be particularly dependent on timely rainfall in low-rainfall climates when irrigation is not available. Therefore, low emergence rate of seeds resulted in the less total biomass of soybean during the most test year. Overall, Huai bean performed the best among the three tested legumes, not only because of its lower ET during the growing season, but also due to its greater improvement in the productivity and WUE in the normal and wet years.

## Conclusions

Water deficiency is the major obstacle to the crop productivity of the dryland agriculture in the Loess Plateau of China. The results from this six-year study confirm that planting the LGM crop (Huai bean, soybean, and mung bean) consumes soil water during the fallow season which can bring varied effects on the following wheat growth and productivity. The effect is positive or neutral when the annual precipitation is adequate, so that there is no significant reduction in the quantity of soil water supplied for the next crop. If this is not the case, the effect is negative. Consequently, it is a viable option to plant the LGMs instead of summer fallow to improve the yield and WUE of wheat and maintain the soil water balance in the normal and wet years. Among the three tested legumes, Huai bean was more suitable than soybean and mung bean under our experimental dryland conditions. Future efforts are needed to find the appropriate field management to alleviate the negative effects of the LGM approach on the next crop in the dry years. These findings will hopefully (i) develop a LGM-based cropping system with a theoretical basis and field management strategies, and (ii) enhance the agricultural productivity, environmental sustainability, and economic profitability, not only in the Loess Plateau of China but also in other similar dryland regions.

## Additional Information

**How to cite this article**: Zhang, D. *et al.* Soil Water Balance and Water Use Efficiency of Dryland Wheat in Different Precipitation Years in Response to Green Manure Approach. *Sci. Rep.* **6**, 26856; doi: 10.1038/srep26856 (2016).

## Figures and Tables

**Table 1:** Probability levels from the analysis of variance (ANOVA) for seasonal evapotranspiration (ET), water productivity (WP), fallow precipitation storage efficiency (PSE), soil water storage during the summer fallow season (SWS), grain yield, evapotranspiration (ET), water use efficiency of wheat (WUE), soil water depletion during the wheat growing season (SWD), and soil water balance (SWB) at a depth of 0–200 cm.

Source	ET_F_^a^	WP	PSE	Yield	ET_G_^a^	WUE	SWS	SWD	SWB
Year (Y)	0.002^**^	0.0058^**^	0.0005^**^	<0.0001^**^	<0.0001^**^	<0.0001^**^	0.0002^**^	0.002^**^	<0.0001^**^
Leguminous green manure (LGM)	0.641^NS^	0.1039^NS^	0.2504^NS^	<0.0001^**^	<0.0001^**^	<0.0001^**^	0.4931^NS^	0.4385^NS^	0.2792^NS^
N fertilizer (N)	–	–	–	<0.0001^**^	<0.0001^**^	<0.0001^**^	–	–	–
LGM × N	–	–	–	0.0403^*^	<0.0001^**^	0.123^NS^	–	–	–
LGM × Y	0.2213^NS^	0.0191^*^	0.1002^NS^	<0.0001^**^	0.0005^**^	<0.0001^**^	0.206^NS^	0.4679^NS^	0.0584^NS^
N × Y	–	–	–	0.042^*^	0.0011^**^	0.0577^NS^	–	–	–
Y× LGM × N	–	–	–	0.7369^NS^	0.0763^NS^	0.7466^NS^	–	–	–

^a^ET_F_ and ET_G_ represent the ET during the fallow season and growing season, respectively. *, **indicate statistically significant differences at P= 0.05, 0.01, respectively (Duncan’s test). NS, not significant.

**Table 2:** ET, WP, and PSE in different precipitation years under LGM treatments in the summer fallow season from 2008 to 2013.

Treatments	ET^a^ (mm)			WP (kg/m^3^)			PSE (%)		
	Dry^b^	Normal	Wet	Dry	Normal	Wet	Dry	Normal	Wet
FW^c^	169 b^d^	265	167	–	–	–	44.9 a	46.4	60.0
HW	206 a	246	178	1.21 a	0.86 b	1.15 a	33.5 b	50.1	57.4
SW	202 a	239	174	0.78 b	0.88 b	1.07 a	34.4 b	51.6	58.1
MW	188 ab	269	186	1.22 a	1.09 a	0.71 b	39.1 ab	45.4	55.2
Average	191	255	176	1.07	0.94	0.98	38.0	48.4	57.7

^a^ET evapotranspiration, WP water productivity, and PSE fallow precipitation storage efficiency.

^b^Dry years include 2008–09, 2009–10, and 2012–13; normal year is 2010–11; wet years include 2011–12 and 2013–14.

^c^The LGM treatments are FW for summer fallow–winter wheat (CK), HW for Huai bean–winter wheat, SW for soybean-winter wheat, and MW for mung bean-winter wheat.

^d^Within the same column, different lowercase letters indicate significant differences at *P* < 0.05 (Duncan’s test) between the LGM treatments.

**Table 3:** Grain yield, ET, and WUE for different precipitation years under the LGM and N fertilizer treatments from 2008 to 2013.

Factors	Yield (kg/ha)			ET (mm)			WUE (kg/m^3^)		
	Dry^a^	Normal	Wet	Dry	Normal	Wet	Dry	Normal	Wet
Leguminous green manure (LGM)									
FW^b^	3109 a^c^	3187 c	5152 b	362 a	339 a	386 a	0.83 ab	0.85 c	1.38 c
HW	2826 b	4427 a	6478 a	311 c	308 b	341 c	0.88 a	1.38 a	2.01 a
SW	2912 ab	3790 b	6576 a	332 b	332 a	368 b	0.87 ab	1.13 b	1.97 b
MW	2676 b	3832 b	5370 b	327 b	320 ab	369 b	0.80 b	1.14 b	1.60 b
N fertilizer (N)									
0	2512 b	3102 b	5195 b	326 b	309 b	343 b	0.75 b	0.93 b	1.55 b
162	3055 a	4074 a	6212 a	333 a	328 a	377 a	0.89 a	1.20 a	1.83 a
ANOVA									
LGM	*	**	**	**	*	**	NS	**	**
N	**	**	**	*	**	**	**	**	**
LGM × N	**	NS	NS	NS	**	**	**	NS	NS

^a^Dry years include 2008–09, 2009–10, and 2012–13; normal year is 2010–11; wet years include 2011–12 and 2013–14.

^b^The LGM treatments are FW for summer fallow-winter wheat (CK), HW for Huai bean-winter wheat, SW for soybean-winter wheat, and MW for mung bean-winter wheat.

^c^Within the same column, different lowercase letters indicate significant differences at *P* < 0.05 (Duncan’s test) between the LGM treatments. *, **indicate statistically significant differences at P = 0.05, 0.01, respectively (Duncan’s test). NS, not significant.

**Table 4:** SWS, SWD, and SWB in the 0–100, 100–200, and 0–200 cm depths in different precipitation years under LGM treatments from 2008 to 2013.

Soil depth (cm)	Treatments	SWS^a^ (mm)			SWD (mm)			SWB (mm)		
		Dry^b^	Normal	Wet	Dry	Normal	Wet	Dry	Normal	Wet
0–100	FW^c^	125 a^d^	149 b	154	147 a	130	141	−22.3 b	19.2	12.2
	HW	101 b	167 a	160	126 b	143	140	−24.4 ab	24.8	20.0
	SW	100 b	164 a	163	129 b	139	144	−28.0 a	24.8	19.1
	MW	111 b	156 b	154	128 b	138	142	−17.3 b	18.8	12.3
100–200	FW	15.5 a	86.2	111	24.4	74.6	100	−8.94	11.5	11.2 b
	HW	3.65 b	83.8	94.2	15.7	75.2	70.5	−12.1	8.62	23.7 a
	SW	3.46 b	88.7	92.6	14.5	84.4	73.7	−11.1	4.29	18.8 ab
	MW	12.7 ab	77.3	94.9	23.9	71.6	75.1	−11.2	5.77	19.8 ab
0–200	FW	140 a	236	264	171 a	205	241	−31.2	30.7	23.4 b
	HW	105 b	251	254	141 b	218	210	−36.5	33.4	43.7 a
	SW	104 b	253	255	143 b	224	217	−39.1	29.1	38.0 ab
	MW	123 ab	234	249	152 b	209	217	−28.5	24.6	32.1 ab

^a^SWS soil water storage during the summer fallow season, SWD soil water depletion during the wheat growing season, and SWB soil water balance.

^b^Dry years include 2008–09, 2009–10, and 2012–13; normal year is 2010–11; wet years include 2011–12 and 2013–14.

^c^The LGM treatments are FW for summer fallow-winter wheat (CK), HW for Huai bean-winter wheat, SW for soybean-winter wheat, and MW for mung bean-winter wheat.

^d^Within the same column, different lowercase letters indicate significant differences at *P* < 0.05 (Duncan’s test) between the LGM treatments.
